# Therapeutically blocking interleukin-11 receptor-α enhances doxorubicin cytotoxicity in high grade type I endometrioid tumours

**DOI:** 10.18632/oncotarget.15187

**Published:** 2017-02-08

**Authors:** Amy Winship, Michelle Van Sinderen, Katarzyna Rainczuk, Evdokia Dimitriadis

**Affiliations:** ^1^ Centre for Reproductive Health, The Hudson Institute of Medical Research, Clayton, 3168, VIC, Australia; ^2^ Department of Molecular and Translational Medicine, Monash University, Clayton, 3800, VIC, Australia; ^3^ Department of Anatomy and Developmental Biology, Monash University, Clayton, 3800, VIC, Australia

**Keywords:** endometrioid, cytokine, chemotherapy

## Abstract

High grade type I endometrial cancers have poor prognosis. Interleukin (IL)11 is elevated in tumours and uterine lavage with increasing tumour grade in women. IL11 regulates cell cycle, invasion and migration and we recently demonstrated that IL11 receptor (R)α inhibition impaired low and moderate grade endometrial tumourigenesis *in vivo*. In this report, we hypothesized that micro-RNA(miR)-1 regulates IL11 and that IL11 promotes high grade endometrial tumour growth. We aimed to determine whether combination treatment using an anti-human IL11Rα blocking antibody (Ab) and doxorubicin chemotherapeutic impairs high grade tumour growth. MiR-1 was absent in human endometrial tumours versus human benign endometrium (*n* = 10/group). Transfection with miR-1 mimic restored miR-1 expression, down-regulated IL11 mRNA and impaired cell viability in grade 3-derived AN3CA human endometrial epithelial cancer cells. AN3CA cell proliferation was reduced in response to Ab and doxorubicin combination treatment versus Ab, IgG control, or doxorubicin alone. Subcutaneous xenograft tumours were established in female Balb/c athymic nude mice using AN3CA cells expressing IL11 and IL11Rα. Administration of recombinant human IL11 to mice (*n* = 4/group) activated IL11 downstream target, signal transducers and activators of transcription (STAT3) and significantly increased tumour growth (*p* < 0.05), suggesting that IL11 promotes high grade tumour growth. IL11Rα blocking Ab reduced STAT3 phosphorylation and combination treatment with doxorubicin resulted in a significant reduction in tumour growth (*p* < 0.05) compared to Ab, doxorubicin, or IgG control. Our data suggest that therapeutically targeting IL11Rα in combination with doxorubicin chemotherapy could inhibit high grade type I endometrioid cancer growth.

## INTRODUCTION

Endometrial cancer is the most common invasive gynaecological malignancy in developed countries, with more than 280,000 new cases occurring on average annually [1]. There is no effective screening test for early detection and there are currently no curative therapies. Alarmingly, the incidence is increasing, particularly in women of reproductive age [2], thought to be attributed to an increase in obesity. Mortality is primarily related to advanced or recurrent disease. Although current radiotherapy or chemotherapy may achieve a transient treatment response, the median survival for these women is less than one year [2].

Type I ‘endometrioid’ carcinoma is the most common type of endometrial cancer, accounting for approximately 85% of cases [3]. Type I endometrial tumours are staged according to the guidelines of the International Federation of Gynecology and Obstetrics (FIGO) [4]. Increasing tumour grade (G1-3) is based histologically on the extent to which the cancer differentiates from normal endometrial morphology by loss of the formation of glandular structures and also has increasing metastatic characteristics. Grading therefore includes the extent to which the cancer invades the uterine corpus and the surrounding peritoneum [3]. Grade 1 cancers are described as well differentiated in terms of their morphology. These cancers are usually confined to the uterus and have good prognosis following surgical intervention and radiotherapy. Grade 2 endometrial cancers are moderately differentiated and display myometrial invasion within the uterus, as well as spread to nearby pelvic and para-aortic lymph nodes. Grade 3 cancer cells are arranged in a haphazard or disorganized way and do not form glands; hence they are described as poorly differentiated and are highly metastatic [4]. Both grade 2 and 3 endometrial cancers have poorer prognosis, compared to grade 1, with metastatic behavior being most closely linked with clinical outcome and cause of death [3].

The cycling endometrium undergoes well-coordinated and regulated processes of proliferation and differentiation in response to estrogen and progesterone respectively. Subsequently, after menopause, the endometrium becomes atrophic. While it is considered that endometrial cancer most frequently arises when the quiescent endometrium is affected by hormonal imbalances, molecular or genetic alterations, or a combination of these factors [3], the precise etiology is poorly understood. Once the critical molecular regulators are discovered, targeted and more effective treatment options may be developed.

Cytokines produced within the tumour microenvironment can promote cancer cell growth, attenuate apoptosis and facilitate invasion and metastasis. Interleukin-(IL)11 is a pleiotropic cytokine that regulates cell cycle, invasion and migration in numerous cell types [5, 6]. IL11 is a member of the IL6 family of cytokines, which also includes leukemia inhibitory factor (LIF), oncostatin M, cardiotrophin-1, ciliary neurotrophic factor, cardiotropin-like cytokine/cytokine-like factor, IL27 and IL31. This family shares a common accessory signalling molecule, glycoprotein (gp) 130, though IL11 signals via its’ own distinct ligand-specific receptor (R) α subunit. This infers non-redundancy between the IL6 family members that share the gp130 signal transducer [7]. In the human endometrium and placenta, IL11 activates the janus kinase and signal transducers and activators of transcription (JAK/STAT3) pathway [8, 9] STAT3 is constitutively active in many human cancers, including endometrial cancer [10] and has the capacity to promote epithelial tumour growth [11].

The expression of IL11 in the female reproductive tract and reproductive cancers has previously been reviewed [12]. In women with endometrial cancer, IL11 levels are elevated in uterine lavage fluid and is positively associated with increasing endometrial tumour grade [13]. IL11 protein [13] and mRNA [14], along with IL11Rα protein levels are progressively increased with increased endometrial tumour grade [13], which is in line with findings in other tumour types such as ovarian and colorectal [15, 16]. Studies have reported pro-tumourigenic roles for IL11 in several epithelial cancers, including breast [17] and colorectal cancer [16] and one recent study highlighted a predominant role for IL11 rather than IL6 in mediating gastric cancer [18]. We have previously demonstrated that IL11 promotes endometrial cancer cell migration *in vitro*, via STAT3 [19] and that blockade of IL11 receptor (R) α reduces grade-1 and grade-2 derived human endometrial epithelial cell line xenograft tumour growth and metastasis in mouse models [20]. However, the regulation and function of IL11 in high grade endometrial cancer cell has not been fully evaluated.

MicroRNA (miR-1) has previously been demsontrated to act as a tumour suppressor in endometrial tumours [21]. IL11 is a predicted target of miR-1 [22]. We hypothesized that miR-1 regulates IL11 in endometrial tumours and that IL11 promotes high grade endometrial tumour growth. We aimed to determine whether combination treatment using anti-human IL11Rα blocking antibody (Ab) and doxorubicin chemotherapeutic impairs high grade tumour growth.

## RESULTS

### MiR-1 is absent in human endometrial cancer and cell lines and miR-1 mimic down regulates IL11 in AN3CA cells

**Figure 1:**
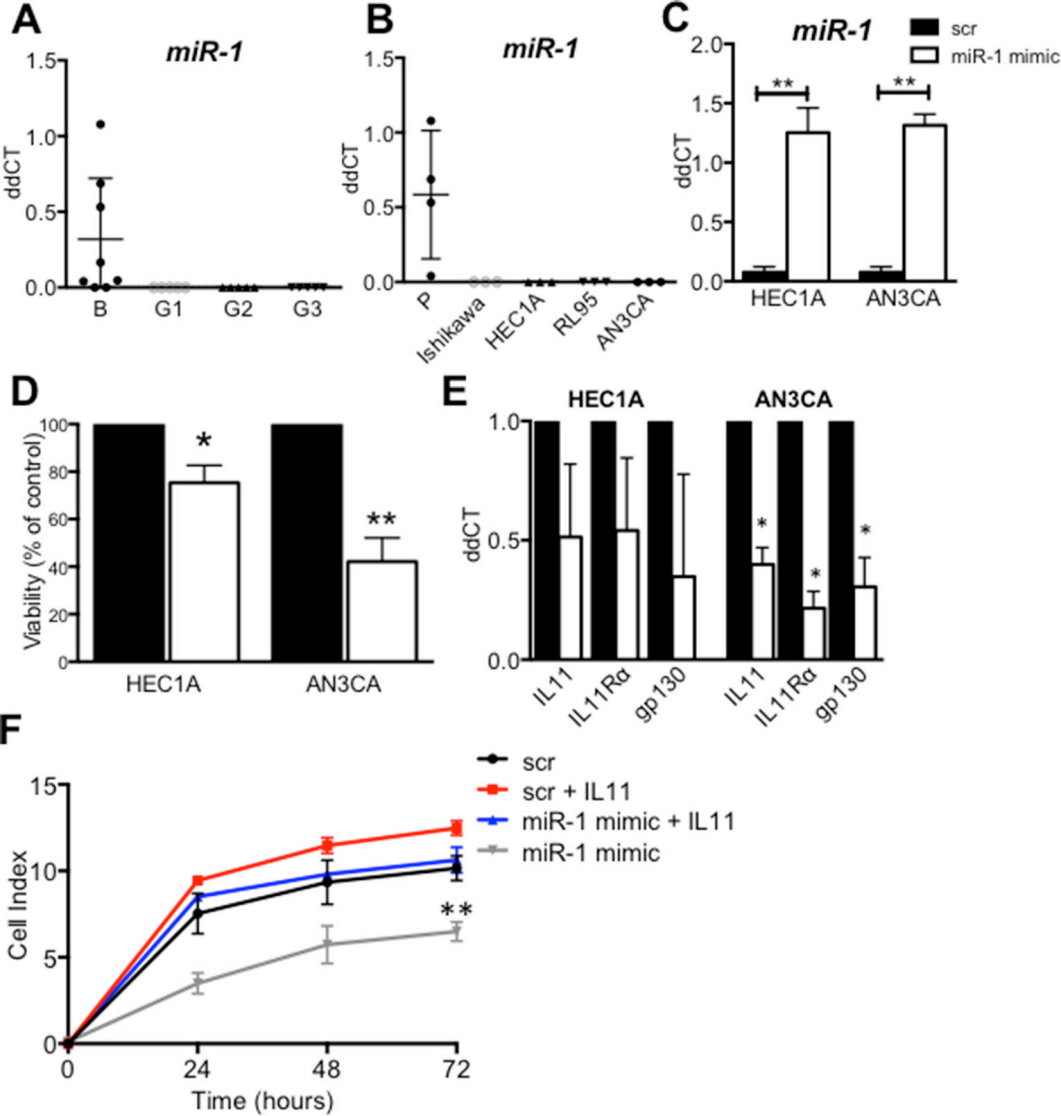
MiR-1 expression and regulation of IL11 in human endometrial cancer and cell lines (**A**) MiR-1 expression was quantified in G1, 2, or 3 human endometrial cancer tissue, or benign (B) endometrium by real-time RT-PCR normalized to snU6 (*n* = 10/group) and in (**B**) normal proliferative phase endometrial epithelial cells (*n* = 4), or human endometrial cancer cell lines; Ishikawa, HEC-1A, RL95 and AN3CA derived from grade 1, 2, or 3 human endometrial cancers respectively, normalized to 18 s (*n* = 3 passages/cell line). (**C**) Transfection efficiency of miR-1 mimic after 72 h in HEC1A and AN3CA cells was confirmed by quantitative real time RT-PCR. (**D**) 72 h post-transfection, the effect of miR-1 mimic or scr control on cell viability was determined by MTT assay (*n* = 3) and on (**E**) IL11, IL11Rα, or gp130 gene expression normalized to 18 s (*n* = 3). (**F**) AN3CA cells transfected with miR-1 mimic or scr control ± IL11 (100 ng/ml) were used in xCELLigence real time proliferation assays performed in triplicate (*n* = 3). Data are mean ± SEM. (C–E) *t*-test, *p < 0.05, **p < 0.01 (F) ANOVA, **p < 0.01.

Quantitative real-time RT-PCR was performed to determine miR-1 expression in whole tissue from G1-3 endometrial tumours versus benign endometrium. MiR-1 expression was detectable in most benign endometrial tissue samples, however expression in G1-3 endometrial tumour tissues was undetectable (*n* = 10/group) (Figure 1A). Similarly, miR-1 was detected in primary human proliferative phase endometrial epithelial cells, but was undetectable in human endometial epithelial carcinoma cell lines (*n* = 3–4/group) (Figure 1B). MiR-1 was overexpressed by transfecting HEC1A and AN3CA cells with miR-1 mimic. MiR-1 was significantly up regulated in both cell types treated with mimic versus scrambled (scr) control (Figure 1C). MiR-1 mimic significantly reduced HEC1A (*p <* 0.05) and AN3CA cell viability (*p <* 0.01) (Figure 1D), and significantly down-regulated *IL11* mRNA and its’ signaling components, *IL11Rα* and *gp130* in AN3CA cells (*p <* 0.05), but not HEC1A cells (Figure 1E). In AN3CA cells, miR-1 mimic significantly reduced cell proliferation versus scr control after 72 h (*p <* 0.01) (Figure 1F). IL11 treatment of scr control AN3CA cells did not significantly alter cell proliferation (Figure 1F). Addition of IL11 to miR-1 mimic transfected cells restored AN3CA cell proliferation to control levels (Figure 1F).

### Anti-human IL11Rα antibody combination treatment with doxorubicin reduces AN3CA cell viability and proliferation *in vitro*

**Figure 2:**
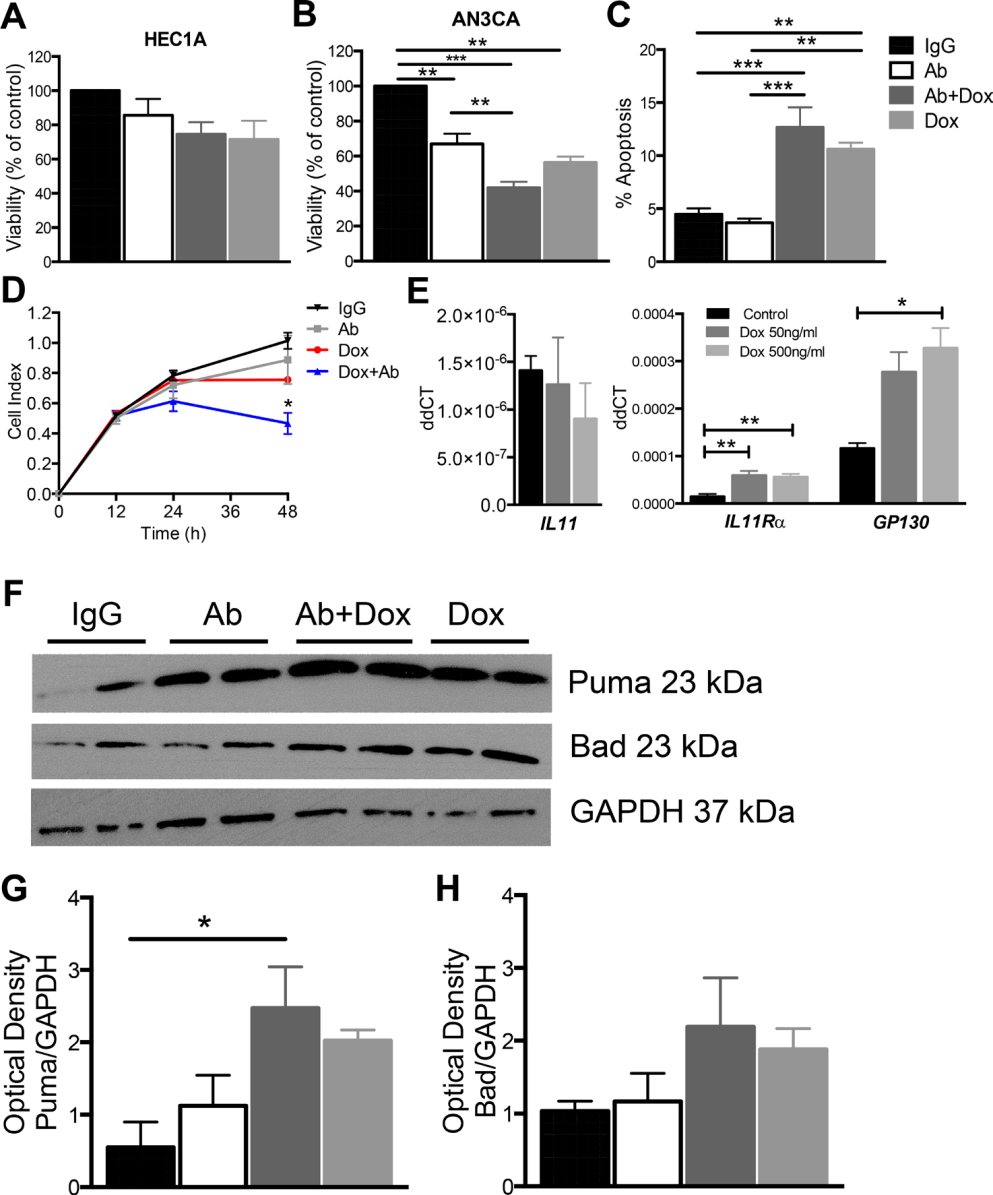
The effect of anti-human IL11Rα antibody combination treatment with doxorubicin on AN3CA cell viability, proliferation and apoptosis *in vitro* MTT assay was performed on (**A**) HEC1A and (**B**) AN3CA cells treated for 24 h with IgG control, IL11Rα Ab (1 μg/ml) ± doxorubicin (500 ng/ml), or doxorubicin alone. (**C**) The effect on AN3CA cell apoptosis was determined after 24 h by flow cytometry and (**D**) real time cell proliferation was analysed by xCELLigence. (**E**) IL11, IL11Rα and gp130 gene expression was assessed in AN3CA cells by quantitative real time RT-PCR in reseponse to vehicle control or doxorubicin treatment (50 or 500 ng/ml) after 6 h. (A–D) Data are mean ± SEM; ANOVA, *p < 0.05, **p < 0.01, ***p < 0.001. (**F**) The effect of IgG control, IL11Rα Ab (1 μg/ml) ± doxorubicin (500 ng/ml), or doxorubicin on pro-apoptotic regulators, Puma and Bad was assessed by Western blot after 24 h treatment, using GAPDH as a loading control (*n* = 3). Densitometry was performed on *n* = 3 blots for (**G**) Puma and (**H**) Bad and normalized to GAPDH. Data are mean ± SEM; ANOVA, *p < 0.05.

HEC1A and AN3CA cell viability was determined in response to treatment with IgG control, IL11Rα Ab alone or in combination with doxorubicin, or doxorubicin alone. There were no differecnes in cell viability in HEC1A cells after 24 h of treatment (Figure 2A). AN3CA cell viability was significantly impaired in response to IL11Rα Ab (*p <* 0.01) and doxorubicin alone (*p <* 0.01), but the greatest effect was seen in response to IL11Rα Ab combination with doxorubicin (*p <* 0.001) and versus IgG control (Figure 2B). Suppression of IL11Rα activity in AN3CA cells by a single dose of the IL11Rα Ab did not alter apoptosis at 24 h (Figure 2C). Doxorubicin treatment (*p <* 0.01) and combination IL11Rα Ab and doxorubicin treatment increased apoptosis (*p <* 0.001) compared to IgG control or IL11Rα Ab alone (Figure 2C). In support, real time cell proliferation analysis revealed a significant reduction in cell proliferation at 48 h in response to combination IL11Rα Ab and doxorubicin treatment (*p <* 0.05) versus all other treatment groups (Figure 2D). Doxorubicin alone did not reduce cell proliferation at 48 h (Figure 2D). To determine whether doxorubicin induces *IL11, IL11Rα* and *GP130* gene expression in AN3CA cells, cells were collected 6 h after doxorubicin treatment. *IL11* mRNA was unchanged, although both *IL11Rα* (*p <* 0.01) and *GP130* (*p <* 0.05) mRNA were significantly increased in response to doxorubicin treatment at 500 ng/ml versus control and *IL11Rα* mRNA was increased in response to a lower concetration of doxorubicin at 20 ng/ml compared to control (*p <* 0.01) (Figure 2E). The effect of IgG control, IL11Rα Ab alone, IL11Rα Ab combination with doxorubicin, or doxorubicin alone on pro-apoptotic regulators was assessed by Western blot after 24 h treatment of AN3CA cells. We examined whether IL11Rα inhibition induced Bad and Puma and could enhance the efficacy of doxorubicin to induce these pro-apoptotic mediators in AN3CA cells. Immunoblotting results showed that IL11Rα Ab combined treatment with doxorubicin significantly increased Puma protein compared to IgG (Figure 2F, 2G). Bad protein was present in in all treatment groups (Figure 2F, 2H).

### IL11 promotes AN3CA xenograft tumour growth *in vivo*

**Figure 3:**
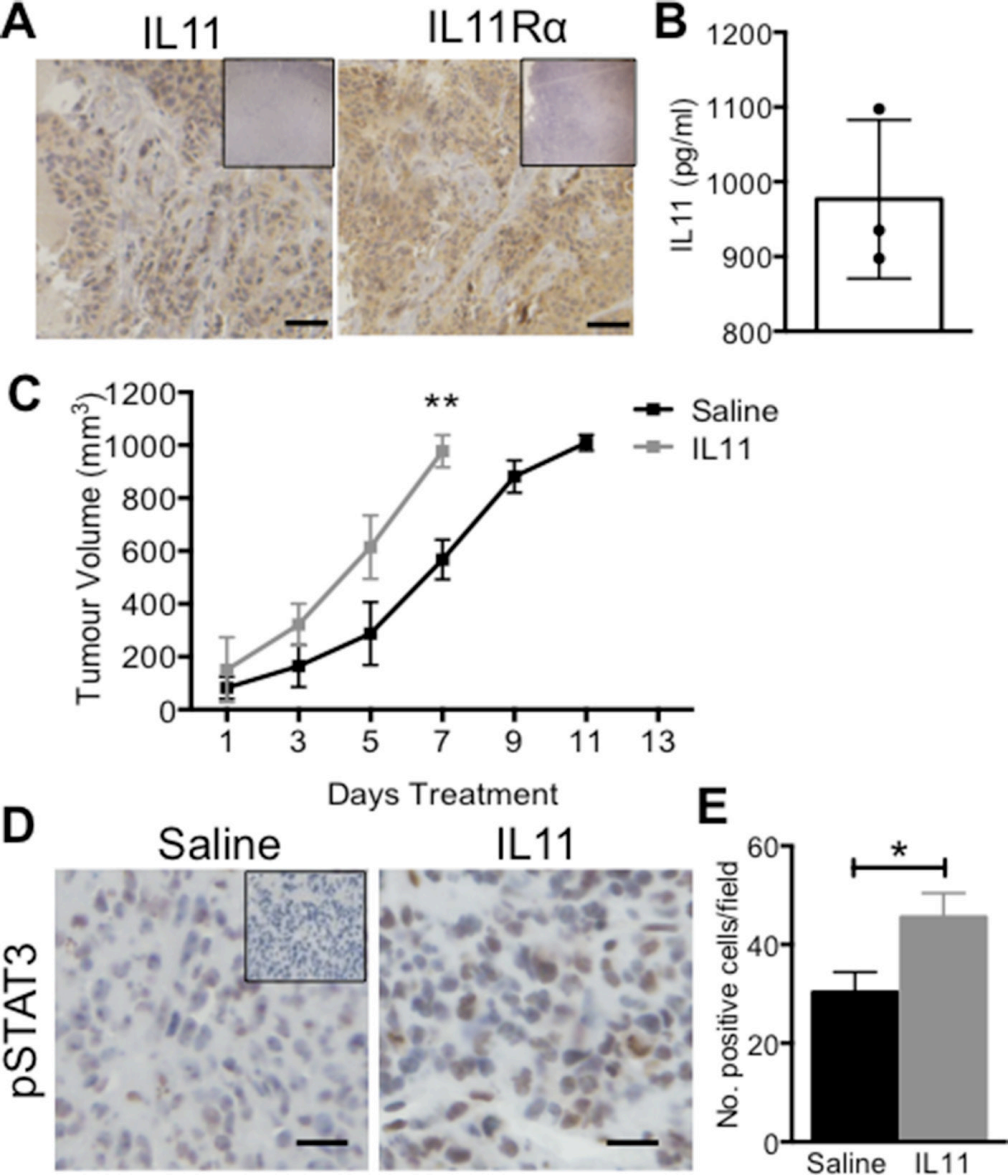
The effect of IL11 on AN3CA xenograft tumour growth *in vivo* Female Balb/c athymic nude mice were inoculated with 5 × 10^5^ AN3CA cells on both hind flanks. (**A**) Immunohistochemistry for IL11 and IL11Rα was performed on untreated AN3CA xenograft tumour tissue sections (*n* = 3). Brown indicates positive staining with blue nuclear counterstain. Bars represent 50 μm. Insets are negative controls. (**B**) IL11 protein (pg/ml) was quantified in ANC3A tumours by ELISA. Data are mean ± SEM of triplicate experiments (*n* = 3). (**C**) Mice with established AN3CA xenograft tumours were administered with saline vehicle control or IL11 (500 μg/kg) three times weekly and tumour volume calculated. (**D**) Representative photomicrographs of pSTAT3 immunohistochemistry on AN3CA subcutaneous control or IL11-treated tumour sections. Bars represent 50 μm. Inset is negative control. (**E**) Total number of pSTAT3 positive cells per field (×20 magnification) were counted from 5 fields per tumour. (D, E) Data are mean ± SEM. *t*-test; *p < 0.05, **p < 0.01 (*n* = 3/group).

To determine whether IL11 facilitates high grade tumour growth *in vivo*, AN3CA subcutaneous xenograft tumours were established in mice. Immunolocalization confirmed that AN3CA tumours produce IL11 and IL11Rα protein in the human-derived tumour epitheilial cells (Figure 3A), suggesting the potential for recombinant human IL11 to signal via IL11Rα in the tumour epitheilium and to target the receptor using our anti-human IL11Rα blocking Ab. Endogenous IL11 protein was also detectable in AN3CA tumour lystaes (976.6 pg/ml ± 61.3) (Figure 3B). Mice with AN3CA subcutaneous xenograft tumours were treated with saline vehicle control or IL11 and tumour volume calculated. IL11 administration resulted in a significant increase in tumour volume by 7 days of treatment, at which point the tumours approached our ethical maximum volume limit (977.25 mm^3^ ± 5.52 versus saline control 567.93 mm^3^ ± 10.11; *p <* 0.01) (Figure 3C). In AN3CA tumour tissue, immunohistochemistry demonstrated that STAT3 is phosphorylated under basal conditions and exogenous IL11 further enhanced this (*p <* 0.05) (Figure 3D, 3E).

### Anti-human IL11Rα antibody combination treatment with doxorubicin reduces AN3CA xenograft tumour growth *in vivo*

**Figure 4:**
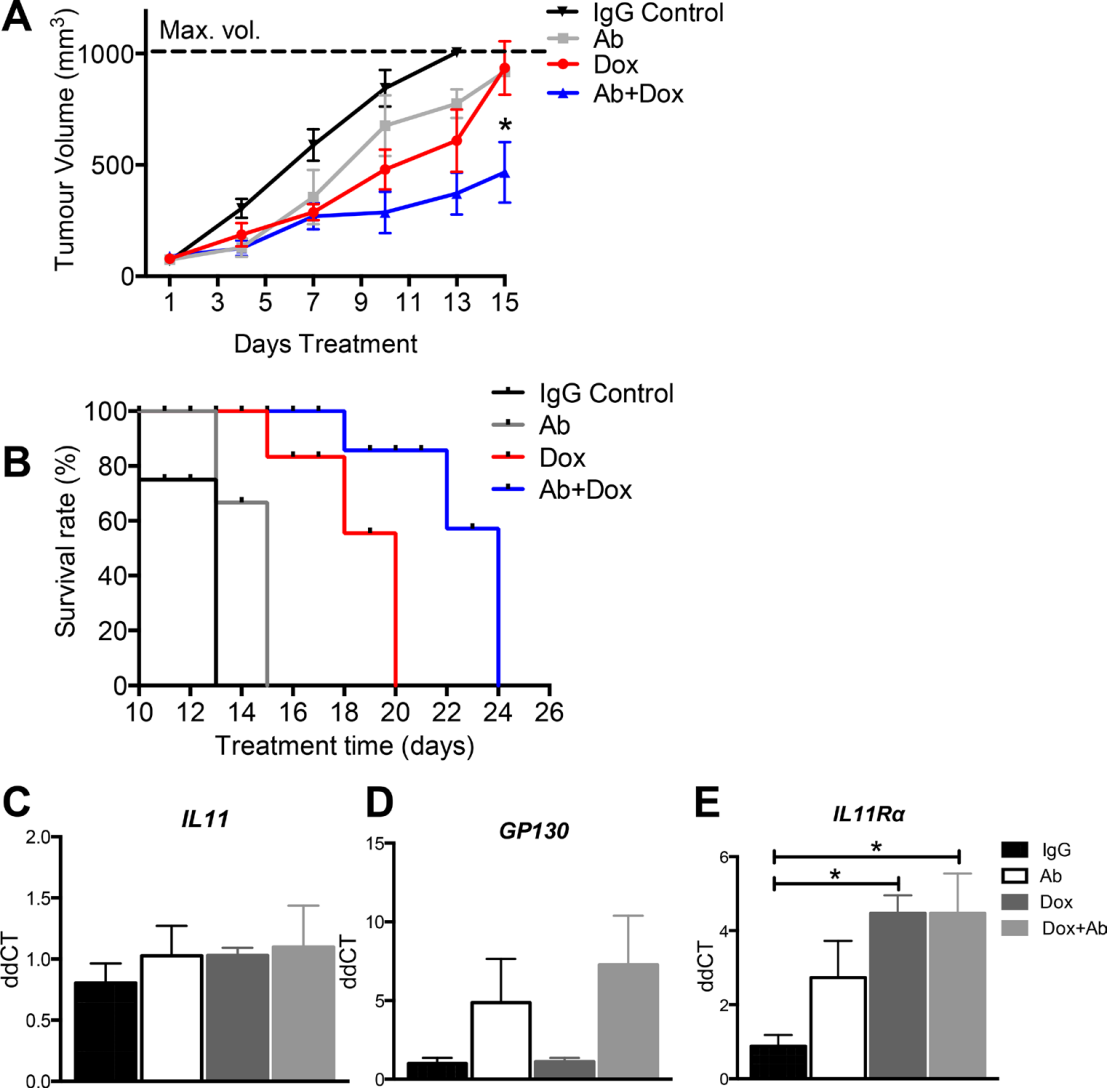
The effect of anti-human IL11Rα antibody combination treatment with doxorubicin on AN3CA xenograft tumour growth *in vivo* (**A**, **B**) Female Balb/c athymic nude mice inoculated with 5 × 10^5^ AN3CA cells on both hind flanks were administered with IgG control, IL11Rα Ab (200 μg per dose 3 times weekly) ± doxorubicin (5 mg/kg once), or doxorubicin alone and tumour volume calculated (*n* = 5 mice/group). (**C**) IL11, (**D**) GP130 and (**E**) IL11Rα gene expression was assessed in AN3CA tumours by quantitative real time RT-PCR. Data are mean ± SEM; ANOVA, *p < 0.05 (*n* = 5/group).

We investigated the potential for IL11Rα inhibition to impair high grade tumour growth *in vivo* and determine whether it may enhance the efficacy of doxorubicin. Mice with subcutaneous AN3CA xenograft tumours were administered with IgG control, IL11Rα Ab alone, or in combination with doxorubicin, or doxorubicin alone and tumour volume calculated. Combination IL11Rα Ab treatment with doxorubicin significantly reduced tumour growth 15 days after the commencement of treatment versus all other treatment groups (466.3 mm^3^ ± 136) (*p <* 0.05) (Figure 4A). Combination treatment enhanced survival before the tumours approached maximum tumour volume at 22 days, versus 13 days for the IgG control group, or 15 days for IL11Rα Ab alone and doxorubicin alone treatment groups (Figure 4A, 4B). IL11Rα Ab treatment did not alter *IL11* mRNA, but led to a trend in increased *GP130* mRNA, while IL11Rα Ab and doxorubicin treatment significantly increased *IL11Rα* (*p <* 0.05) in subcutaneous AN3CA tumours (Figure 4C–4E).

### Anti-human IL11Rα antibody reduces activated STAT3 and combination treatment with doxorubicin induces apoptosis in AN3CA tumour xenograft tumours *in vivo*

**Figure 5:**
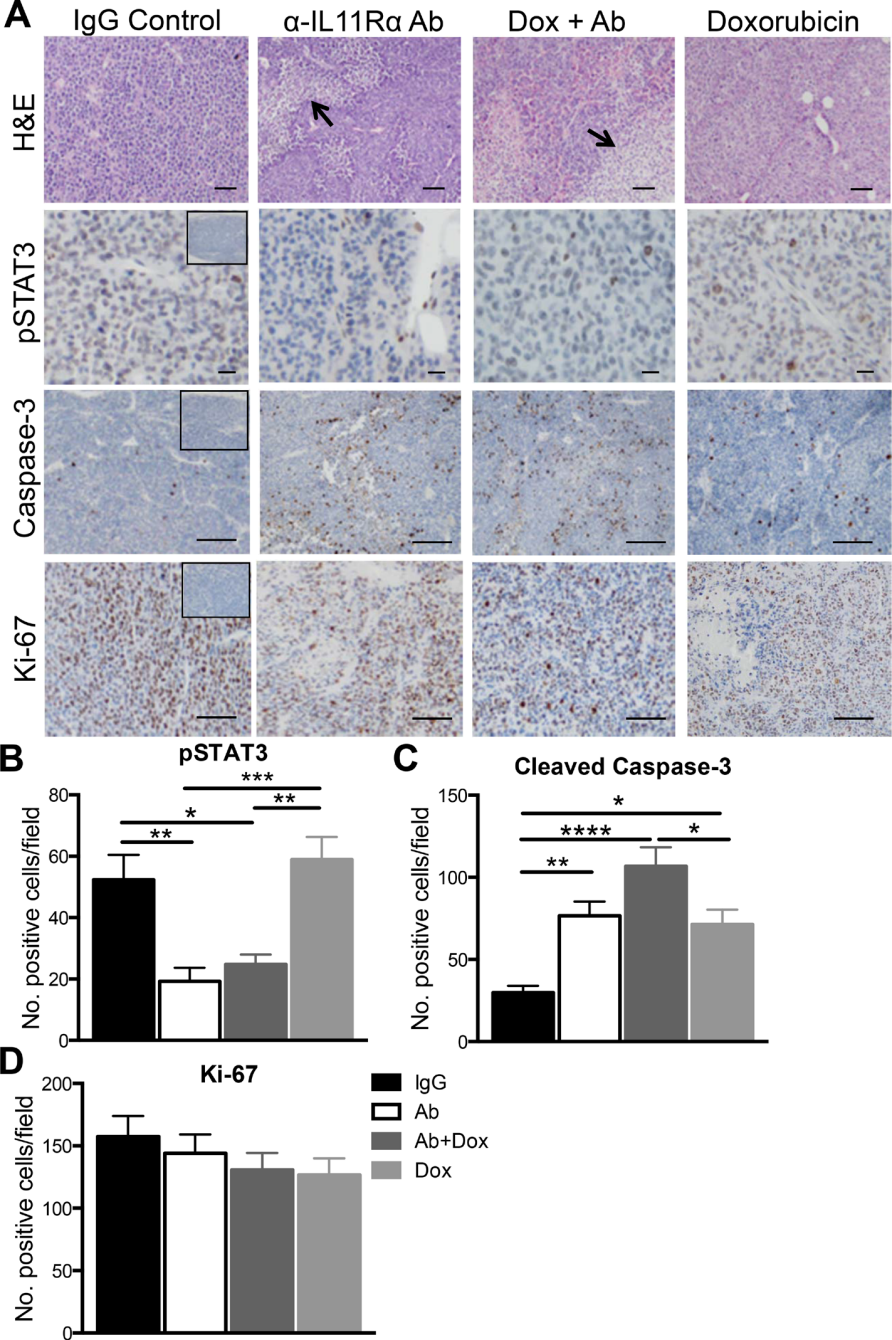
The effect of anti-human IL11Rα antibody combination treatment with doxorubicin on AN3CA xenograft tumour morphology *in vivo* (**A**) Representative photomicrographs of hematoxylin and eosin stained AN3CA subcutaneous tumour sections are shown in the top panel, arrows denote areas of tissue necrosis. AN3CA subcutaneous tumour sections were immunostained for pSTAT3, cleaved-caspase-3 (apoptosis marker) and Ki-67 (proliferation marker). Bars represent 100 μm (H&E), 50 μm (pSTAT3) and 200 μm (caspase-3, Ki-67). Insets are negative controls. The total number of (**B**) pSTAT3, (**C**) cleaved caspase-3 and (**D**) Ki-67 positive cells per field (×20 magnification) were counted from at least 5 fields per tumour. Data are mean ± SEM. *t*-test; *p < 0.05, **p < 0.01 (*n* = 5/group).

Hematoxylin and eosin staining showed areas of tissue necrosis in AN3CA subcutaneous tumour tissues treated with IL11Rα Ab alone or in combination with doxorubicin. Activated pSTAT3 was evident in tumours treated with IgG control or doxorubicin alone, but levels were significantly reduced in the epithelial cells of tumours treated with IL11Rα Ab alone or in combination with doxorubicin (Figure 5A, 5B). Semi-quantitation of cleaved-caspase-3 immunostained tumour tissue sections indicated a significant increased in tumour epithelial cell apoptosis in response to IL11Rα Ab or doxorubicin treatment alone (*p <* 0.01) compared to IgG control. This treatment effect was enhanced in response to combined treatment of IL11Rα Ab with doxorubicin versus IgG (*p <* 0.001), or doxorubicin alone (*p <* 0.05) (Figure 5A, 5C). There were no significant differences in the number of Ki-67 positive proliferating cells between treatment groups (Figure 5A, 5D).

## DISCUSSION

We recently demonstrated that IL11Rα inhibition reduces low and moderate G1-derived Ishikawa and G2-derived HEC1A endometrial xenograft tumour growth and metastasis in ectopic and orthotopic mouse models [20]. We and others previously determined that IL11 mRNA and protein are up-regulated in endometrial tumour tissue and uterine lavage in women [13, 14] and we have shown that IL11 alters endometrial cancer cell function *in vitro*, via STAT3 [19]. In this report, we investigated the regulation and function of IL11 in high grade, G3-derived AN3CA endometrial epithelial cells *in vitro* and *in vivo* in xenograft tumours in mice. MiR-1 down-regulated *IL11* gene expression and its’ signaling components, *IL11Rα* and *gp130* in G3-derived AN3CA endometrial epithelial cells. Exogenous IL11 promoted AN3CA subcutaneous xenograft endometrial tumour growth *in vivo* andIL11Rα inhibition and doxorubicin combinantation treatment enhanced apoptosis in AN3CA cells *in vitro* and impaired high grade endometrial tumourigenesis *in vivo*.

IL11 is crucial for normal female reproductive physiology in humans and mice [12]. In the cycling human endometrium, prostaglandin E(_2_) regulates IL11 [23]. IL11 is regulated by hypoxia [24] and oxidative stress [25] in other epithelial tumour types. PGE_2_ and hypoxia synergize to promote endometrial adenocarcinoma cell proliferation [26], mediated by the calcium-calcineurin nuclear factor of activated T cells (NFAT) pathway in G1-derived Ishikawa cells and primary human endometrioid cancer tissue explants [14]. Here, we investigated the regulation of IL11 by miR-1 in HEC1A and AN3CA cells. MiRs are short, highly conserved non-coding RNA sequences that regulate the expression of an estimated 50% of genes in the human genome, by binding to specific mRNAs via targets located in their 3′ UTR [27, 28]. Their main role is post-transcriptional regulation by binding to sequences of specific mRNAs to cause translational arrest or degradation of the target mRNA [28].

MiR-1 is reported to act as a tumour supressor, since restoration of mature miR-1 impairs endometrial cancer cell migration and invasion [21]. MiR-1 has approximately 1000 predicted targets in different cell types [22, 29], with only phosphodiesterase 7A (PDE7A) experimentally confirmed in endometrial cancer cells [21]. *IL11* is a predicted gene target of miR-1 [22] and miR-1 transfection of Hela cells down-regulates *IL11, IL11Rα* and *STAT3* mRNA [29]. Loss of miR-1 expression in primary type I human endometrial cancer [21] correlates with elevated IL11 expression [13, 14]. Similarly, we found loss of miR-1 expression in a panel of human endometrial epithelial cell lines compared to normal proliferative phase endometrial epithelial cells. Restoration of miR-1 by synthetic miR-1 mimic transfection significantly down-regulated *IL11*, *IL11Rα* and *gp130* in AN3CA cells, but not HEC1A cells. This difference may be attributed to the fact that predicted miR-target gene interactions are cell type and context dependant. Regardless, miR-1 mimic significantly reduced cell viability in both HEC1A and AN3CA cells, in line with findings in other endometrial epithelial cell lines, including G1-derived HEC1B and G2-derived HEC265 [21]. In AN3CA cells, miR-1 mimic reduced cell proliferation and addition of IL11 to miR-1 mimic transfected cells restored AN3CA proliferation to control levels. This finding suggests that miR-1 may regulate proliferation in endometrial cancer cells at least in part via IL11, however, the direct binding of miR-1 with the 3′UTR of IL11 was not established.

This is the first study to determine a functional role for IL11 in mediating high grade endometrial tumourigenesis *in vivo*. Compared with other endometrial epithelial cell lines, AN3CA xenografts form tumours rapidly. Interestingly, despite the detection of endogenous IL11 in AN3CA tumours,, exogenous IL11 administration enhanced tumour growth even further. We found constituitve STAT3 activation, indicated by nuclear pSTAT3 immunostaining in control tumours, but increased pSTAT3 positive tumour epithelial cells in tissues from mice treated with IL11. IL11Rα inhibition reduced STAT3 activation in tumours, with, or without doxorubicin combination treatment. In addition to the JAK/STAT pathway, IL11 can signal via the mitogen activated protein kinase/extracellular signal-regulated kinase (MAPK)/(ERK), or the phsophotidylinositol-3 kinase (PI-3K) pathways [7]. Doxorubicin has also been shown to alter AKT and ERK activity in Ishikawa, RL95-2 and KLE andometrial cancer cell lines [30, 31], although this has not been investigated in AN3CA cells. We previously demonstrated that exogenous IL11 phosphorylates STAT3, but not ERK, or AKT in Ishikawa, HEC1A and AN3CA cells *in vitro* [19] and Ishikawa and HEC1A xenograft tumours *in vivo* [20], confirming that IL11 likely does not act via these pathways in these cells. Based on these data, we limited our investigations to IL11 induced STAT3 signaling, although a role for doxorubicin and IL11Rα combination treatments in mediating ERK and AKT signaling is possible.

In contrast to the transient activation of STAT3 in normal cells, many tumours exhibit constitutive STAT3 activity. STAT3 activation promotes survival and prevents tumour cell apoptosis in numerous cancer types [32]. Blockade of IL-6-induced STAT3 in hepatocellular carcinoma cell lines facilitates doxorubicin-mediated apoptosis in previously doxorubicin-resistant cells [33]. Similary, STAT3 inhibition using a JAK inhibitor increased doxorubicin sensitivity in human metastatic breast cancer cells [34]. However, STAT3 signalling is required for normal cell cycle regulation throughout the human body. Therefore, targeting cytokines that induce STAT3, could avoid off-target side effects of total STAT3 or JAK inhibition. IL6, another STAT3-activating cytokine, has been of much interest and antibodies that neutralize IL6 or block IL6 receptor (R) α are in clinical trials for ovarian, prostate, and renal cancers [35, 36]. In an orthotopic nude endometrial carcinoma model, IL6 promoted tumour growth [37], while targeted inhibition of the IL6 receptor dramatically reduced tumour growth in HEC1A cell-derived subcutaneous xenografts in immune-deficient mice [38].

IL11 is now also emerging as an attractive therapeutic target for cancer. In the recent first in human study, six human prostate cancer patients were treated with a site-targeted peptidomimetic drug that targets IL11Rα, which increased apoptosis in bone metastatic lesions [39]. A possible side effect of blocking IL11Rα in cancers in humans, is the effect on platelet counts, since IL11 is used as a treatment option for thrombocytopenia after chemotherapy [40]. However intriguingly, no thrombocytopenia was observed in the prostate cancer trial in men [39], or in a gastric cancer mouse model [18].

We recently demonstrated that an IL11Rα blocking Ab reduced Ishikawa and HEC1A cell proliferation, invasion and migration *in vitro* and also impaired tumour growth *in vivo* [20]. Our findings from the present study demonstrated that IL11Rα blockade alone was not sufficient to induce AN3CA cell apoptosis *in vitro* or impair proliferation or tumourigenesis *in vivo*. Interestingly, doxorubicin treatment alone, or combination with IL11Rα inhibition treament did not alter cell proliferation, assessed by cell count analysis from Ki67 immunohistochemistry stained tissue sections at the study endpoint. It is possible that a single dose of doxorubicin administered to mice at day 1 of the treatment study was no longer efficacious in halting cell proliferation after 15 days. Despite no reduction in tumour cell proliferation rate, IL11Rα Ab and doxorubicin combination treatment resulted in reduced xenograft tumour growth *in vivo* versus all other treatment groups.

It is well known that IL11 has an anti-apoptotic role [25] and here we demonstrated that IL11 signalling blockade induced apoptosis in endometrial tumour xenografts. Considerable evidence suggests that IL11 is also required for epithelial cell proliferation and survival, leading to the initiation and progression of cancer in the gastric mucosa and colon in humans and mice [18]. IL11 regulates cell cycle and cell survival targets, via the STAT3 pathway, including cyclinD3, p21, Bcl-xL, Bcl-2 and survivin [18, 41]. Therefore, we chose to examine additional targets, including two well-established pro-apoptotic regulators, Puma and Bad. Puma and Bad were not altered by treatment with an IL11 Ra mutein antagonist in a mouse model of gastric cancer [18], however we demonstrated their induction in response to IL11Rα Ab combination treatment with doxorubicin chemotherapy. This finding suggests that IL11Rα inhibition may facilitate doxorubicin-mediated apoptosis in AN3CA cells, that were previously doxorubicin resistant.

Our findings are supported by a number of studies in other epithelial-derived tumour types, where impaired IL11Rα signalling reduced gastric [18] and prostate cancer growth and metastasis [42], further strengthening the rationale for therapeutically targeting IL11Rα in endometrial cancer. IL11 was recently implicated in experimental models of chronic inflammation-associated tumourigenesis, mediated at least in part by over activation of STAT3 [43]. Additionally, IL11 and IL11Rα overexpression are associated with tumour progression, growth and differentiation and poor prognosis in colorectal [16], gastric [43], hepatocellular [44] and breast [45] cancers. Furthermore, a recent study using a breast cancer cell line (MDA-MB-468) xenograft model showed that IL11 expressing sub-clone populations in heterogeneous tumours acted in a non-cell autonomous manner, causing non IL11-expressing cells to behave in the same manner to drive proliferation and tumour growth [46]. Together these findings highlight an emerging and predominant role for this cytokine in promoting tumourigenesis.

In conclusion, IL11 has comprehensively emerged as an important factor stimulating epithelial endometrial tumour progression and IL11Rα inhibition may offer a new strategy for novel therapeutic development.

## MATERIALS AND METHODS

### Ethics statement

Written informed consent was obtained from each patient and the study was approved by the Southern Health Research and Ethics Committee (#09317B) at Monash Medical Centre Melbourne, Australia.

### Endometrial cancer patient samples

RNA from type I human endometrioid cancer grades (G)1, 2 and 3, or benign endometrium (*n* = 10/group) whole tissue was obtained from the Victorian Cancer Biobank (Project #13018) (Table 1).

**Table 1:** Clinical characteristics of the patients used in this study

Patient no	Age	Menopausal status	Cancer Grade	%MI
1	65	Post	1	0
2	56	Post	1	29
3	84	Post	1	80
4	34	UK	1	0
5	78	Post	1	4
6	55	Post	1	0
7	51	UK	1	0
8	77	Post	1	21
9	48	UK	1	0
10	78	Post	1	12
11	73	Post	2	38
12	52	UK	2	UK
13	60	Post	2	18
14	88	Post	2	73
15	63	Post	2	100
16	54	UK	2	38
17	61	Post	2	UK
18	60	Post	2	27
19	75	Post	2	73
20	71	Post	2	49
21	54	Post	3	38
22	59	Post	3	33
23	77	Post	3	25
24	59	UK	3	UK
25	68	Post	3	13
26	55	UK	3	81
27	64	Post	3	49
28	62	Post	3	52
29	71	Post	3	19
30	66	Post	3	UK

Abbreviations: Post: post-menopausal; UK: unknown; % MI: % myometrial invasion.

### Endometrial tissue collection

Endometrial biopsies were collected by curettage from women with regular menstrual cycles throughout the menstrual cycle. Biopsies were obtained from women with known normal fertility during the proliferative phase of the menstrual cycle (*n* = 4). All women had regular menstrual cycles of 26–32 days, were not using intrauterine contraceptives and had not used hormones for at least 3 months prior to surgery. Biopsies were examined by an experienced gynaecological pathologist to confirm that they showed no evidence of possible endometrial dysfunction. All biopsies were histologically dated according to the Noyes criteria [47].

### Primary human endometrial epithelial isolation

Endometrial epithelial cells were prepared as previously published [48]. Briefly, endometrial tissue was digested with collagenase and the suspension was filtered through 43 and 11 mm nylon mesh to collect endometrial epithelial glands. The cells and epithelial fragments were collected and resuspended in a 1:1 mixture of Dulbecco's modified eagle's medium (DMEM)/Hams F-12 (Gibco) supplemented with 10% foetal calf serum (FCS; Invitrogen), and 1% antibiotic–antimycotic solution (Gibco, Auckland, NZ) and plated. A purity of 95% was necessary for the cells to be used experimentally.

### Cell lines and culture

G2-derived HEC1A and G3-derived AN3CA endometrial epithelial cell lines were purchased from the American Type Culture Collection (ATCC, 2013) and cultured in McCoy's medium, or DMEM medium respectively with 10% FCS. Monash Health Translational Precinct Medical Genomics authenticated the cell lines in June 2016.

### RNA preparation and quantitative real time RT-PCR

RNA was extracted from cultured cells or whole xenograft endometrial tumour tissue using Tri Reagent (Sigma) according to the manufacturer's instructions. Isolated RNA was reversed transcribed into complimentary DNA with M-MLV RT system (Life Technologies) by using the TaqMan primer sets for miRs (Applied Biosystems) or Oligo primers (Sigma) for non-miRs. Real time PCR was performed using the TaqMan Fast Universal PCR Master mix (Applied Biosystems) or Power SYBR Green master mix (Applied Biosystems) by using TaqMan probes or specific primer pairs (Table 2). MiR-1 expression levels were normalised against control snU6 probes. Gene expression was normalised against 18 s.

**Table 2:** Primer sequences

**IL11**	F 5′-GTTTACAGCTCTTGATGTCTC-3′
	R 5′-GAGTCTTTAACAACAGCAGG-3′
**IL11Rα**	F 5′-GTCCCCTGCAGGATGAGATA-3′
	R 5′-AGGCCAAGGCAAGAGAAGAT-3′
**p130**	F 5′-CATAGTCGTGCCTGTGTGCT-3′
	R 5′-GCCGTCCGAGTACATTTGAT-3′

### Micro-RNA (miR-1) mimic transfection

HEC1A or AN3CA cells were transfected according to manufacturers instructions using Lipofectamine^®^ RNAiMAX and miR-1 mimic (100 nM; Life Technologies) for 72 h. A scrambled microRNA sequence (scr) (Life Technologies) was used as a control.

### IL11Rα blocking antibody and doxorubicin chemotherapeutic

Anti-human IL11Rα blocking antibody (Ab) and IgG control were provided by CSL Ltd, Parkville, Australia. Doxorubicin hydrochloride was purchased from Sigma-Aldrich.

### MTT cell viability assay

HEC1A or AN3CA cells transfected with miR-1 mimic or scr control were seeded at a density of 10,000 cells per well in 96-well flat-bottom microplates (Costar, USA) 72 h after transfection. HEC1A or AN3CA cells seeded at a density of 10,000 cells per well were serum starved for 12 h, then treated with IgG control, or IL11Rα Ab (1 μg/ml) [20], doxorubicin (500 ng/ml), or Ab and doxorubicin and cultured for another 24 h. MTT (Sigma Aldrich) was added to each well in 10 μl (5 mg/ml in PBS) and incubated at 37°C for 5 h. Following the incubation period the medium was aspirated and 200 μL of acidified isopropanol was added per well. The medium was pippetted to dissolve crystals. The cells were incubated on the rocking platform at room temperature for 10 mins. The absorbance of the sample was determined at a wavelength of 560 nm on an automatic Wallace Envision plate reader (Perkin Elmer). The absorbance values of the treated cells were compared with the values generated from the control cells and reported as the percentage viability of control.

### xCELLigence real time functional assays and cell treatments

Experiments were carried out using the RTCA DP xCELLigence instrument (Roche), which was placed in a humidified incubator maintained at 37°C with 95% air/5% CO_2_. Cells were seeded in E-plate 96 at 10,000 cells/well in 5% FCS medium and the plate was monitored once every 15 min for a total of 72 h. AN3CA cells transfected with miR-1 mimic or scr control for 72 h were treated with/without recombinant human IL11 (100 ng/ml) (R&D systems) [20] at the time of seeding for xCELLigence real time proliferation assays. In subsequent experiments, AN3CA cells were treated with IgG control, or IL11Rα Ab (1 μg/ml), doxorubicin (500 ng/ml), or Ab and doxorubicin. Data was calculated using RTCA software 1.2, supplied with the instrument (ACEA) and exported for statistical analysis.

### Flow cytometry and cell-cycle analysis

AN3CA cells were cultured in serum free medium for 24 h to synchronize populations into G0. Medium was replaced with complete media containing 10% FCS, and cells treated with IgG control, or IL11Rα Ab (1 μg/ml) doxorubicin (500 ng/ml), or Ab and doxorubicin. Cells were harvested after 24 h and fixed overnight in 70% ethanol. Cells were stained with FxCycle PI/RNase staining solution (Molecular Probes) and analyzed on a BDFACSCanto II flow cytometer. Cell cycle and apoptosis analysis and model fitting was performed with FlowJo (FlowJo LLC).

### SDS-PAGE and western blotting

Cells were lysed in ice-cold lysis buffer (50 mM Tris-HCl (pH 7.5), 150 mM NaCl, 2 mM EDTA, 2 mM EGTA, 25 mM NaF, 25 mM β-glycerolphosphate, protease inhibitor cocktail (Calbiochem) and the protein was quantified by the BCA assay. Equal protein per sample was resolved on 8–10% sodium dodecyl sulfate (SDS)–polyacrylamide gel electrophoresis (PAGE) gels, transferred to polyvinyldifluoride (PVDF) membranes (GE Healthcare Bio-Sciences), blocked with 5% non-fat dry milk in Tris-buffered saline (TBS) containing 0.1% Tween-20 (Bio-Rad) and probed with polyclonal antibodies against Bad (D24A9) rabbit mAb (1:1000; Cell Signaling Technology #9239), or Puma (D30C10) rabbit mAb (1:1000; Cell Signaling Technology #12450) overnight at 4°C, followed by three wash steps. Membranes were incubated for 1 h at room temperature with secondary anti-rabbit Ig-horseradish peroxidase (HRP) linked, (1:5000; DakoCytomation) and signals were developed with enhanced chemiluminescence detection system reagent (Pierce). Membranes were stripped and incubated with anti-GAPDH (1:5000; Cell Signaling Technology #8884) as a protein loading control. Membranes were exposed to autoradiography film (Hyperfilm ECL; GE Healthcare) for between 10 sec and 1 min. Films were scanned and densitometry was performed and normalized to GAPDH loading control, using Image J Software.

### Animals

Animal experiments were conducted in female, 5–7 week old, athymic, BALB/c nude mice purchased from Animal Resources Centre; Western Australia, housed in specific pathogen-free conditions, with food and water available *ad libitum* and held in a 12 h light and dark cycle. Use of all animals was in accordance with the guidelines of the Monash Medical Centre Animal Ethics Committee under Ethics Approval number MMCB/2012/07.

### Subcutaneous tumour inoculation

AN3CA cells were resuspended in serum free medium at a concentration of 5 × 10^6^ cells/ml. Both flanks of each animal were inoculated with 100 μl (5 × 10^5^ cells). Once palpable, tumours were measured with digital calipers (Hare & Forbes Machinery House) and tumour volume calculated using the following formula: (length × width^2^)/2 (mm^3^) [20].

### Animal treatments and tissue collection

Once subcutaneous tumours were palpable and measured 80–100 mm^3^, mice were randomized into groups and administered by intraperitoneal injection with saline vehicle control, or recombinant human IL11 (500 μg/kg) three times weekly (*n* = 3/group). For therapeutic studies, mice were administered with doxorubicin once (5 mg/kg), either alone, or in combination with 200 μg IL11Rα Ab three times weekly, or IL11Rα Ab or control IgG alone for three weeks, or until tumour volume approached a maximum limit of 1000 mm^3^. At the completion of the study, primary tumours were dissected in half and either snap frozen, or fixed in 4% paraformaldehyde for 24 h.

### Histology and immunohistochemistry

Histologic confirmation of uterine tumour formation was performed using hematoxylin and eosin (H&E) staining. For immunohistochemistry, formalin-fixed subcutaneous endometrial tumour sections (4 μm) were dewaxed in histosol (2 × 10 min), rehydrated in ethanol and antigen retrieval performed in 0.01M sodium citrate (pH 6) before endogenous peroxidase activity was quenched with 3% hydrogen peroxide in methanol for 10 min. Non-specific binding was blocked with 10% normal goat serum and 2% normal human serum, in Tris Buffered Saline (TBS) for 30 min. IL11 (H-169) (Santa Cruz #7924; 1:100), IL11Rα (Novus Biologicals #62351; 1:100), Cleaved caspase-3 (Asp175) (Cell signaling #9661; 1:500), phosphorylated (p)STAT3 (Tyr705) (Cell signaling #9145 s; 1:100), or Ki-67 (Abcam ab16667; 1:500) antibody was applied overnight at 4°C. Negative control isotype rabbit IgG (Dako) was included for every tissue section. Antibody localization was detected by sequential application of biotinylated goat anti-rabbit IgG (1:200) for 30 min followed by Vectastain ABC Elite kit (Vector) for 30 min. Peroxidase activity was visualized by the application of diaminobenzidine substrate (DakoCytomation). Tissues were counterstained with Harris hematoxylin (Sigma–Aldrich) and mounted. Using CellSense software, eight photographs at 20X magnification were taken from each tumour section representing more than 90% of the tumour cross section. A blinded observer counted the numbers of pSTAT3, cleaved caspase-3, or Ki-67 positive cells. The values from each field were averaged and expressed as number of positive cells/field for each tumour.

### Enzyme linked immunosorbent assay

Levels of IL11 protein were quantified in untreated control AN3CA subcutaneous tumour lysates using a human IL11 ELISA (#ELH-IL11; RayBiotech) according to the manufacturer's instructions.

### Statistical analysis

Statistical analysis was carried out using GraphPad Prism (GraphPad Software 6.0) and data assessed by Student's *t*-test for two groups. Multiple groups were compared using 1-way ANOVA, with Tukey's post-hoc test. Results of *p <* 0.05 were considered statistically significant.
